# Contemporary full-mouth rehabilitation using a digital smile design in combination with conventional and computer-aided design/manufacturing restorative materials in a patient with bruxism: A case report

**DOI:** 10.1097/MD.0000000000018164

**Published:** 2019-11-27

**Authors:** Jae-Hyun Lee, Sung-Hun Kim, Jung-Suk Han, In-Sung Luke Yeo, Hyung-In Yoon

**Affiliations:** a Department of Prosthodontics, One-Stop Specialty Center, Seoul National University Dental Hospital; b Department of Prosthodontics and Dental Research Institute, School of Dentistry, Seoul National University, Seoul, South Korea.

**Keywords:** bruxism, digital dentistry, digital smile design, full-mouth rehabilitation, tooth wear

## Abstract

**Rationale:**

Full-mouth rehabilitation of patients with bruxism and severely worn dentition poses a great challenge to clinicians. Several treatment planning methods and restorative materials are used to treat tooth wear in modern dentistry. Clinicians should be able to select the most suitable treatment planning methods and materials for individual patients depending on their specific situation.

**Patient concerns:**

A 47-year-old male was referred for evaluation of a severely worn dentition.

**Diagnoses:**

Clinical and radiographic evaluation revealed tooth wear in the entire dentition. The interocclusal distance at rest was 4 mm, and the patient had a parafunctional habit of bruxism.

**Interventions:**

A digital smile design was used to formulate a treatment plan. Full-mouth rehabilitation was performed using a combination of conventional and digital materials and methods.

**Outcomes:**

The full-mouth restoration showed satisfactory functions and esthetics. No complications were observed in the restorations, supporting tissues, and temporomandibular joints during 2-year follow-up.

**Lessons:**

In clinical practice, it is important to determine the optimal combination of the available methods for treatment planning. This case report details the formulation of a unique treatment plan for the dental rehabilitation of a severely worn out dentition, which is considered challenging due to the limitations imposed by biological tissues and restorative materials. The use of conventional and digital tools for treatment planning, patient education, and treatment execution was demonstrated.

## 1 Introduction

The development of digital dentistry is accelerating with the advancement and use of computer-aided design and computer-aided manufacturing (CAD/CAM) in daily dental practice.^[1,2]^ With regard to digital dentistry, the use and development of tooth-colored restorative materials has been increasing in conjunction with CAD/CAM technology and esthetic demands of patients.^[3]^ Consequently, the use of traditional noble metals and their alloys as restorative materials is rapidly decreasing.^[4]^ Although many new restorative materials such as glass ceramics have been developed for use in digital dentistry, they have limited ductility and integrity compared with conventional precious metal restorations.^[5]^ Ductility and malleability play important roles in evenly distributing the stress applied to the restoration, preventing excessive force from being transmitted to the teeth and periodontal tissues.^[6–8]^

Patients who exhibit wearing off of the entire dentition have long been considered a restorative dental challenge.^[9,10]^ Once the enamel is worn out by a parafunctional habit, such as bruxism, the dentin is exposed, and abrasion proceeds rapidly.^[11]^ In these patients, a full-mouth rehabilitation procedure is indicated. There have been several recent clinical reports on full-mouth rehabilitation using a variety of new CAD/CAM restorative materials, such as zirconia and lithium disilicate.^[12,13]^ Moreover, there have been several reports on digital diagnosis and planning methods.^[14,15]^

However, there have been few studies investigating the different combinations of conventional and digital diagnostic methods and materials. The different advantages of conventional and digital methods make a combination of these methods a viable option and represent the best of both worlds in contemporary dental practice. Hence, this clinical report describes a full-mouth rehabilitation procedure in a patient with bruxism using a combination of conventional and CAD/CAM restorative materials with a digital smile design (DSD) concept. We have also outlined the considerations for selecting adequate planning methods and restorative materials.

### 1.1 Consent statement

The patient has provided informed consent for the publication of this case report and accompanying images.

## 2 Case report

**Figure 1:**
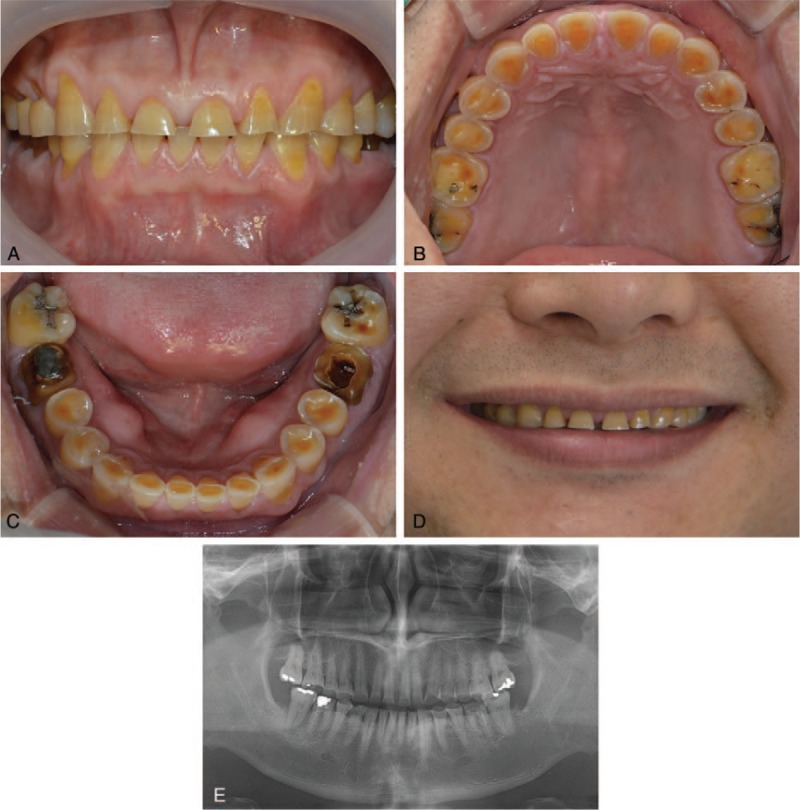
Patient presentation before treatment. Severe wear of the entire dentition is observed on (A) facial, (B) maxillary occlusal, (C) mandibular occlusal, (D) smile views, and (E) panoramic radiograph.

A 47-year-old Asian man was referred by a local clinic to the Department of Prosthodontics to examine wear of the entire dentition. The patient was concerned about tooth structure loss and was dissatisfied with his smile owing to the short clinical crown length of the anterior teeth. A review of his medical history revealed that he had been diagnosed as a hepatitis B virus carrier. The patient had a parafunctional habit of bruxism and clenching. Intraoral and radiographic examination revealed severe attrition and dentinal exposure of the entire dentition. The mandibular right second molar exhibited vertical mobility, and his mandibular angle was highly developed, as seen on a panoramic radiograph. The patient had a >20-year history of tooth wear. The interocclusal distance at rest was 4 mm (Fig. 1).

**Figure 2:**
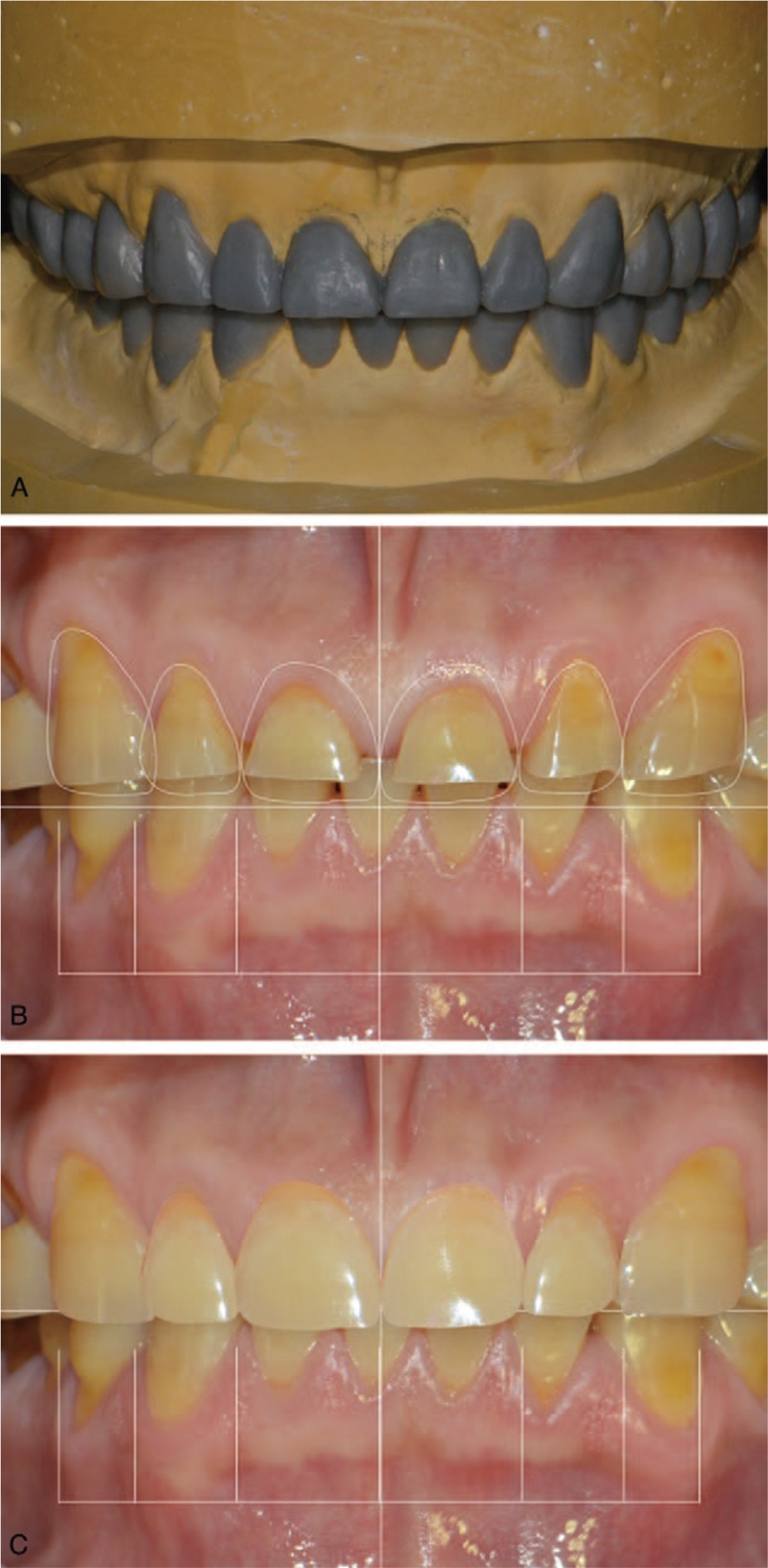
Combination of conventional and digital diagnostic methods. (A) Conventional diagnostic waxing was useful for simulating treatment outcomes and fabricating provisional restorations. (B, C) Digital smile design was useful for presenting outcome to patient.

A diagnostic wax-up was prepared on the casts mounted on a semi-adjustable articulator (Hanau Modular Articulator System, Whip Mix Corp, Louisville, KY). After the occlusal vertical dimension was elevated by 2 mm with respect to the incisal guide pin of the articulator, a full-mouth diagnostic wax-up was created. DSD was also performed using digital presentation software (Keynote, iWork, Apple Inc, Cupertino, CA, USA) to merge the simulated shape of the definitive crowns with the facial photo of the patient. This had the added benefit of open and easy communication with the patient. The upper border of the lower lip was used as the reference line for the maxillary incisal curve to restore the smile line. Adequate levels and locations of the zeniths of the maxillary anterior teeth were simulated on the program. Subsequently, the digitally designed shapes of the anterior teeth were presented to the patient who confirmed that he was satisfied with the simulated treatment outcome (Fig. 2).

Interim full-mouth crown restorations were fabricated with acrylic resin (Jet, Lang Dental Manufacturing Co., Wheeling, IL, USA) by duplicating the diagnostic wax-up. The interim restorations were made to imitate the teeth shapes and smile line of the simulated outcomes of the DSD analysis.

**Figure 3:**
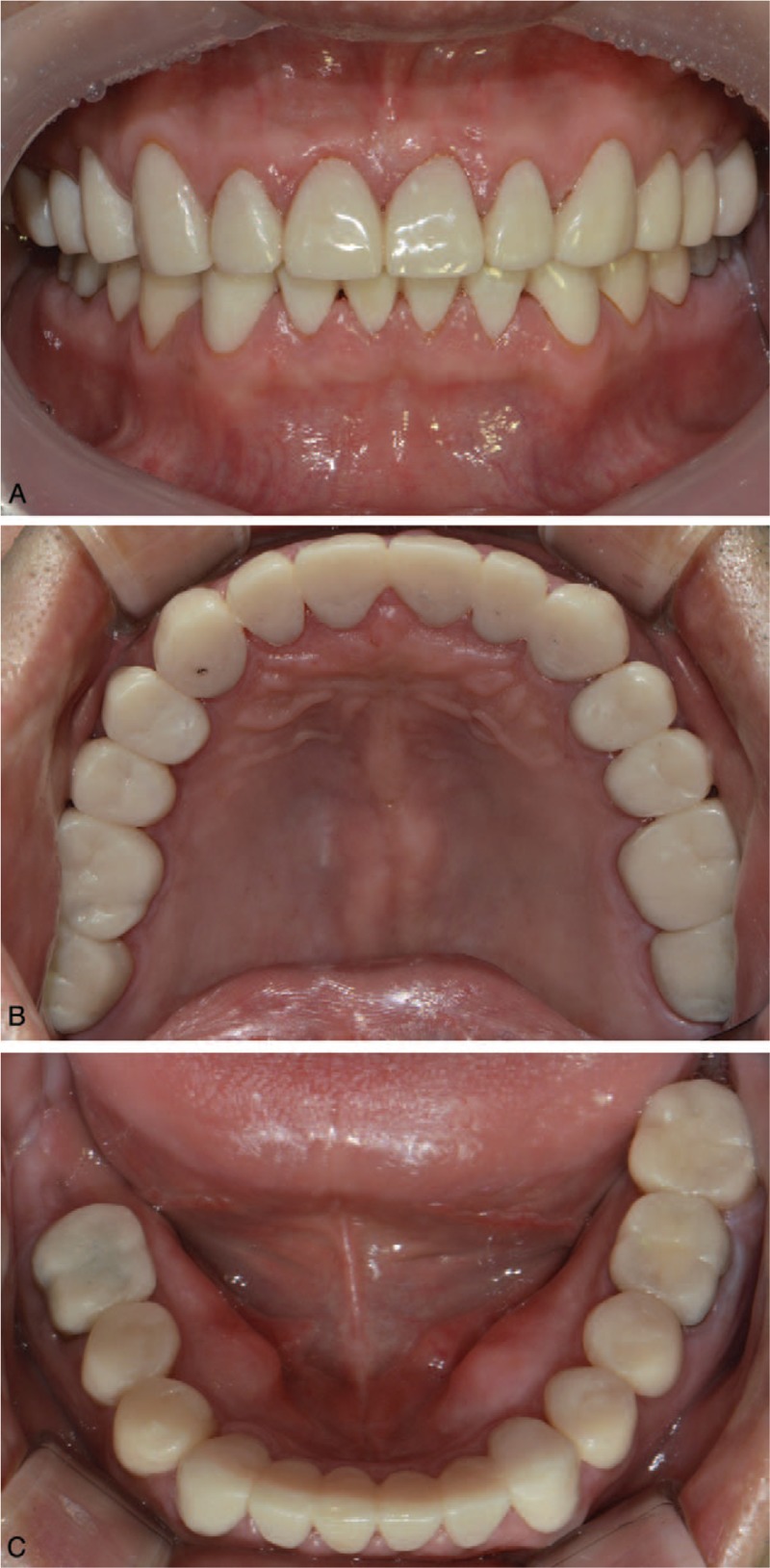
Provisional restorations. Provisional restorations verifying planned tooth position, vertical dimension of occlusion, and mandibular movement on (A) facial, (B) maxillary occlusal, and (C) mandibular occlusal views.

The clinical crown-lengthening procedure of maxillary incisors was performed with guidance from the vacuum shell guide made from the diagnostic wax-up. The shells of the interim crown restorations were relined with acrylic resin following crown preparation of the entire dentition (Fig. 3). Botulinum toxin (Botulax, HUGEL, Inc., Chuncheon-si, Gangwon-do, South Korea) was injected into both masseter muscles to reduce the masticatory force and parafunctional habit. During the 8-month follow-up, the patient appeared to have adapted well to the increased occlusal vertical dimension without any complaints of muscle or joint pain.^[16]^

In consideration of the patient's bruxism and wear history, specific restorative materials for the definitive prostheses were selected carefully. The plan was to use a combination of conventional precious metal alloy restorations and digital CAD/CAM restorations. The maxillary second molar, mandibular first molar, and second molar were to be restored with conventional cast gold crowns. The maxillary first and second premolars and first molar were to be restored with porcelain-fused-to-gold (PFG) crowns, wherein the occlusal surface was made of cast gold, and only the buccal surfaces were veneered with porcelain. The mandibular first and second premolars were to be restored with monolithic zirconia crowns because the occlusal surfaces of these teeth are esthetically relevant. The maxillary and mandibular anterior teeth were to be restored with conventional PFG crowns.

The definitive impressions of the maxilla and mandible were obtained using vinyl polysiloxane (Honigum, DMG, Hamburg, Germany). The master casts were fabricated with Type IV dental stone (GC Fujirock EP, GC Europe N.V., Leuven, Belgium). Cross-mounting was performed on the semi-adjustable articulator, and the casts duplicated the intraoral provisional crowns. Definitive restorations were made by replicating the shape of the provisional crowns, which were tried and finalized in the oral cavity. Monolithic zirconia crowns (BruxZir, Glidewell Laboratories, Newport Beach, CA, USA) were fabricated using the digital method using data from the three-dimensional scans of the provisional crowns. In addition, the gold and PFG crowns were made by conventional gold casting and the porcelain build-up method.

**Figure 4:**
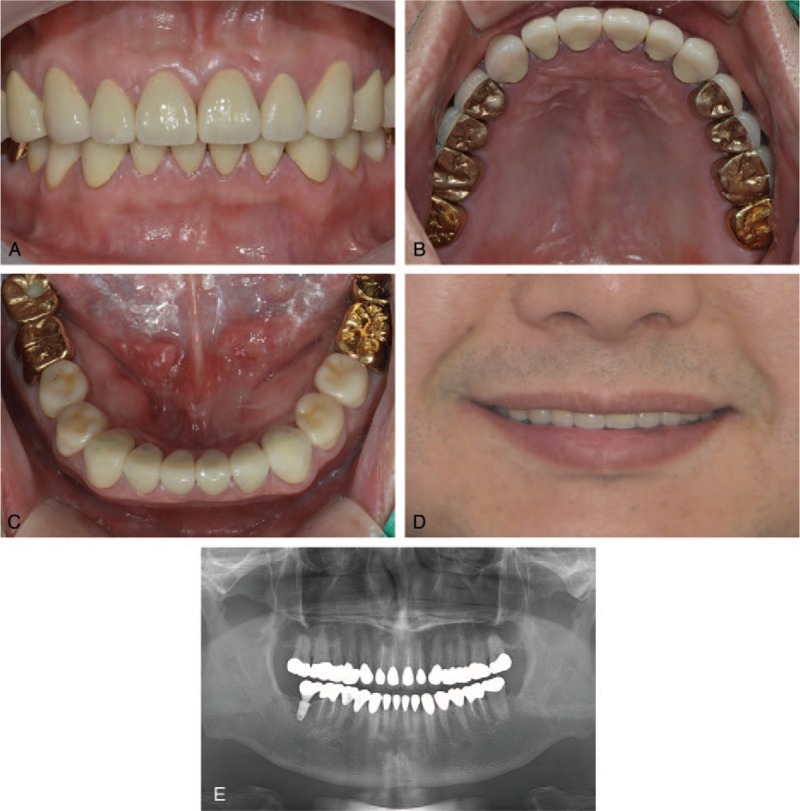
Contemporary combination of conventional and digital full-mouth restorations and postoperative situation. (A–C) Cast gold crowns on maxillary second molar and mandibular first and second molars. Porcelain-fused-to-gold crowns with gold occlusal surface on maxillary first molar and maxillary premolars. Monolithic zirconia crowns on mandibular premolars. Porcelain-fused to-gold crowns or porcelain-fused-to-zirconia crowns on maxillary and mandibular anterior teeth. (D) The resulting smile is similar to the presented digital smile design plan. (E) Panoramic radiograph acquired at the 2-year follow-up shows good treatment outcome.

The gold and PFG crowns were cemented with resin-modified glass ionomer cement (GC FujiCEM 2, GC, Tokyo, Japan), and the monolithic zirconia crowns were bonded with self-adhesive resin cement (RelyX U200, 3 M ESPE, St. Paul, MN, USA). A night guard was made for the patient who used it for 1 month. During the follow-up period of 2 years, the full-mouth restorations were well maintained and no complications were observed in the restorations, supporting tissues, or temporomandibular joints (Fig. 4).

## 3 Discussion

A diagnostic wax-up can be examined from various aspects in three-dimensions and is generally considered a more practical diagnostic method for full-mouth rehabilitation or anterior esthetic restoration than DSD, which uses two-dimensional photographs. Prosthodontists can analyze the occlusion in the centric and eccentric mandibular positions, three-dimensionally, on a diagnostic wax-up positioned on an articulator. They can subsequently replicate it in the form of provisional and, eventually, definitive prostheses. Prosthodontists are accustomed to visualizing the result of the diagnostic wax-up on a patient's face based on several years of experiences; however, this is more difficult for patients. Thus, when consulting with patients, it is advantageous to use the digital techniques such as DSD, which simulate and visualize the shape of the definitive prosthesis superimposed on the patient's photograph. In this manner, digital and conventional methods, used in parallel, substantially simplify the diagnostic process. Although dental three-dimensional computer-aided design (CAD) programs can simulate the diagnostic wax-up digitally, there have been a few studies on the eccentric jaw movement of virtual articulators in CAD programs, which state that they should be used carefully in patients with full-mouth rehabilitation.^[17]^

In the selection of restorative materials, we carefully considered the patient's history of severe wear on the entire dentition. First, the restoration of the occlusal surfaces of all teeth using cast gold was considered, which has been used successfully for approximately a century.^[18]^ The maxillary second molars and mandibular first and second molars were restored with conventional cast gold crowns, as they receive a relatively large amount of occlusal force but are not esthetically relevant.^[7]^ The occlusal surfaces of the mandibular premolars were considered esthetically important because they are often visible during mouth opening. Therefore, we believe it is necessary to restore the occlusal surfaces of the mandibular premolars with monolithic zirconia crowns without veneers to prevent fractures of the veneer porcelain on the occlusal surfaces under load. A PFG crown with a gold occlusal surface was selected as the restorative material of the opposing maxillary premolars to the monolithic zirconia crowns, which can better tolerate occlusal impact, owing to the ductility and malleability of cast gold; thus, it can retain the esthetics of the porcelain veneer on the buccal surface.^[6,8]^

In the present clinical report, we determined that PFG and porcelain-fused-to-zirconia (PFZ) crowns have great potential as restorative materials for the anterior teeth. Noble translucent monolithic zirconia materials may be a favorable option for the anterior teeth of patients with less stringent esthetic needs. However, maximizing the esthetic satisfaction via porcelain veneers was critical for the current patient who was relatively young (47 years). Concerns have been raised with regard to the higher fracture rate of PFZ crowns than of PFG crowns.^[19–21]^ Therefore, we consider PFG crowns to have a slight advantage as a restoration material for the anterior oral cavity of this patient; additionally, this material was expected to better endure the lateral force associated with this patient's severe bruxism.

## 4 Summary

Although it is important to focus on the recent advances in digital dentistry and clinical research, we should consider conventional restorative materials and methods that have been used and studied over many decades. An adequate combination of conventional and digital methods appears to be the best treatment option in contemporary dental practice. This clinical report describes the use of conventional and digital methods in the planning of treatment and selection of restorative materials for a patient with severe bruxism, which yielded excellent functional and esthetic results.

## Author contributions

**Conceptualization:** Jae-Hyun Lee, Sung-Hun Kim, Jung-Suk Han, In-Sung Luke Yeo, Hyung-In Yoon.

**Data curation:** Jae-Hyun Lee.

**Formal analysis:** Jae-Hyun Lee.

**Methodology:** Jae-Hyun Lee.

**Project administration:** Jae-Hyun Lee, Sung-Hun Kim, Jung-Suk Han.

**Resources:** Jae-Hyun Lee.

**Software:** Jae-Hyun Lee.

**Supervision:** Sung-Hun Kim, Jung-Suk Han.

**Validation:** In-Sung Luke Yeo, Hyung-In Yoon.

**Visualization:** Jae-Hyun Lee.

**Writing – original draft:** Jae-Hyun Lee.

**Writing – review & editing:** Jae-Hyun Lee, Sung-Hun Kim, Jung-Suk Han, In-Sung Luke Yeo, Hyung-In Yoon.

Jae-Hyun Lee orcid: 0000-0002-2631-7722.
